
JOURNAL INFORMATION
==============================
NLM Title Abbreviation: Atlanta Med Surg J (1884)
NLM Journal ID: 101251623

Atlanta Medical and Surgical Journal (1884)

Publisher: Biomedical Journal Digitization Project

ARTICLE INFORMATION
==============================
PMCID: PMC9201842
PMID: 35829599
Article version: 1
Subjects: Selections and Abstracts

Selections and Abstracts

Print publication date: 1899 Jan
Volume: 15
Volume Series: New Ser
Issue: 11
First page: 772
Last page: 792
License: This work is free of known copyright restrictions. For more information, please see PMC Back Issue Digitization.
License URL: https://creativecommons.org/publicdomain/mark/1.0/

==============================

### SELECTIONS AND ABSTRACTS.

### Bill Nye and the Nurses.

I have just been sent to the hospital for twenty days.

My physician did it.

He did it with an analysis.

Anybody who amounts to anything nowadays gets analyzed.

Sometimes you find casts, sometimes you find maple sugar, and sometimes you find acids, oxides, paint, oils, varnish, white lead, borax, albumen, lime, hair and cement. In these cases the patient should be placed under a strict diet, or he will, in the course of his life, become a corpse.

I go into details about this, because a false impression got out a few weeks ago to the effect that I came here for another purpose. A reporter came to see me and I sent word to him that I was then out on the operating-table in such a position that I could see no one, while an elderly surgeon was engaged in removing a porous plaster received during the war.

I was not serious in saying this, but unfortunately I have the reputation for absolute veracity and seriousness, so that the statement got into the papers as *bona fide,* and caused American securities to go down two points in one day.

I like it very much here.

*Saturday.*—Another little cripple boy named Charlie comes afternoons to play with me. We have a set of building blocks and can make most anything.

Yesterday the Chicago Ladies' Society for the Prevention of Good came to see us and gave me a kiss and a red apple.

The lady who kissed me was the vice-president of the society, and hurt me when she strained me against her breast-pin. I hate a woman who cannot control herself that way.

I went and took a dose of medicine to take the taste out of my mouth.

To-morrow we have an operation here in my ward. A large lady from La Salle will be removed from a Hungarian tumor.

The medical students will be here and see the operation. The best student for 1894, who has been neither absent nor tardy, will get the tumor.

I have to diet very closely, or I may gradually have a diabetic tendency. I get a slice of toast every day and a thermometer.

The rules are very strict. Patients are requested not to die in the house.

Some of the nurse-girls are perfectly lovely. They are very pretty and kind-hearted. I like to have one of them hold me up in my little cot while I am drinking my koumiss at night.

*Sunday, 3 p. m.*—An analysis to-day shows more casts, fibrin, gelatin, and some zinc and copper. The chemist also discovers that in 1853 I fell from an apple tree and tore my panties in two places. He also says that I will be unhappy with my third wife. She will be unhappy also.

Last evening we had a concert and operation at the hospital. Yesterday afternoon the North Side Ladies' Society for the Prevention of Good paid us a visit. I crawled under the covers and remained in a rigid attitude, giving them a sort of stiff, as a partially idiotic friend of mine said afterward.

The general secretary spoke as she passed by something about "kissing him for his mother," but as she started to raise the sheet from my face I bit her a little in sportive mood, and she gave a shrill and echoing scream. I do not mind being an invalid, but charitable societies must not press me to their bosom. It hurts.

*Monday, 4 p. m.*—Temperature two-fifths of one degree above normal. Pulse regular, but sluggish. Have got all my business arranged, even to terms for shipment home. Ate scraped raw beef for breakfast, using rear quarter of Colorado steer, with pepper and salt on same. Acute gastritis seems to be one of the features of disease. I have to eat simple things like raw bullocks and keg oysters.

Another chemical and microscopic analysis was made yesterday of sputum, showing traces of nicotine and other poisons. Adieu, kind friends, I'm going home. A sweet young novice, who is training for a nurse, took my pulse this A. M. Took quite a while to find it, but I did not murmur or repine. I am trying to learn to love everybody, for to that bourne to which my chemist says that I am going, I should carry with me no enmities, no bitterness. Ta, ta, vain world, begone—

Ah, here comes the other rear breadth of the bullock! Pass me the salt, please, and see that my grave is kept occasionally squirted on during the dry weather. I have left a small fund for the purpose.

The life here at the hospital is delightful, and while I am fading away it is a joy to have loving hands bathing my little hot footies and manicuring my knobby brow.

The Stockyards' Flower Mission paid us a visit this A. M. and left a big, wet cauliflower on each breast.

One large porterhouse lady, with a blue badge on her flank laid a big egg-plant on my pillow, and when I sung out cheerily, "Cut, cut, cudat cut!" she cursed me bitterly and called me a great, coarse thing.

She was right. I ought not to frolic on the crumbling edge of a long, dank grave.

Good-bye, wicked world. After December you will have to pay your own taxes, so the chemist says, for traces of one lung, also floating island and ice cream were found in this last analysis. Do not mourn for me, kind friends, and choke and sob and make yourselves sick. It will be vain. Just live as I have done, so that you may come where I am at. Live upright lives and run the lawn mower about every ten days o'er my humble grave during the summer. That is all you can do. Weep not. In me you have lost a man who can never be replaced, but never mind—the world will have to drag on somehow. I couldn't be here all the time. Anybody with a particle of sense must have seen that I couldn't live forever.

---

While penning the above words a messenger boy has come swiftly in with a note from the chemist. He says in his note:

"We regret that an error was made in your case by our assistant, who, in the rush of business here at the college, has got your analysis somewhat confused with that of the justly celebrated race horse, Nancy Hanks. We unfortunately got the sputa mixed. On going over your case again, we find that, whereas there are signs of glanders in the Hanks analysis, you are, as a matter of fact, almost too healthy. You have phenomenal health and seem free from tendency to pleuro-pneumonia, thrush and epizootic, while in the Hanks case there are tubercles present and signs of bots.

"Abstain from pie in large quantities and avoid night air. We hardly know what to suggest for you to use in place of the night air, after dark, but you must not use night air. Wear heavy Norwegian woolen socks during the cold weather and do not think too much. You are prone to overthink yourself. Go carefully in this direction and you will live to be a burden to your friends. Wishing you good fortune and regretting the confusion of your case with that of Hanks, we beg to enclose our bill and to wish you well."

So to-day I leave my kind little nurses in their sweet and neat attire. Good-bye, girls, I'm going home where they know me. No one there will count my fevered pulse in the still watches of the night. No one there will put a nice hot-water bag, that feels like a Mexican hairless dog, at my feet.

I wring the hand of the superintendent with reluctant haste, and leave the hospital with regret at 2 P. M.

Seriously, what a blessing it is, when we are weary of work and the gastric functions go on a sympathetic strike and the solar plexus goes away and sits down on a stone pile to weep over the situation; when you are in that state of pneumogastric flunk where even you have to threaten your digester with arrest, and liver sulks in a corner, and Old Man Gastric buttons up the coating of your stomach and looks sour, what a godsend that one can go to one of these cosy corners, out of the current of whoop and hurrah, and eat raw steak and be sort of made much of.

---

### NERVE FATIGUE OF SCHOOL CHILDREN.

Dr. R. C Sneidt, of the Franklin and Marshall College, presented a voluminous address (*Public Health*, July) on the hygiene of school-attendance, before the fifth annual meeting of the Associated Sanitary Authorities of Pennsylvania (*vide Journal*, June 18, 1898, p. 1469), in which he urged an ampler protection of the health of the overworked children of the public schools. He argues against the afternoon session as being unnecessary confinement, if the pupils have a long and properly arranged morning session. The following are some of his conclusions:

1. I am convinced that the esthesiometer is the proper instrument for the measurement of fatigue.

2. My tentative experiments with American boys prove a superior normal strength of their mental vigor under favorable conditions of the atmosphere.

3. Unfavorable conditions of the atmosphere show an unusually large percentage of abnormal fatigue of the nervous system.

4. I attribute this condition to the utter lack of systematic open-air exercise.

5. I advocate such exercises for children as a prime necessity, because the foundations of a healthy and useful life are laid between the seventh and fifteenth years, but never afterward.

6. Training of all the functions of the physical organism should precede instruction, because it will develop the necessary mental strength.

7. Children should be classified according to their individual characteristics, and stress should be laid upon the development of their weakest organs, in such a way, however, as to leave plenty of room for individualization.

8. Such exercise should take place every afternoon, and should be continued until the limit of normal fatigue is reached.

9. The exercises should be grouped into muscular, cutaneous and respiratory.

10. All these exercises should be essentially exercises of the nervous system, to lead to a proper development of character.

The writer holds that it is not learning, primarily, but health and character should be the watchword of our schools; first, training, then instruction, the sequence of a rational educational policy. Pedagogics, as a science, should therefore no longer be based upon philosophy, but upon physiology and hygiene. The chief factor in every school board should be the well-trained physician, and the physician only. His investigations carried on for a number of years, have led him to the firm conviction that the American boy is the superior of the boy of any other nation on the face of the globe; he is worthy of special considerations because his training will in the end produce the superior man, for only during the years of boyhood, from the seventh to the fourteenth, fifteenth and sixteenth year, can training be made successful; if neglected then it will forever remain neglected. Considering the large number of heterogeneous branches now taught in our public schools, and, at the same time, the intense interest in all sorts of ill-directed physical exercises manifested by both girls and boys, we conclude that the endurance for every specific activity can be but small, while the sum total of all the energies consumed is rather larger than in former ages; however, the mere external exhibition of ability is, in the case of children, not by any means the absolute criterion for the sum total of living forces within the human organism. Let us abolish afternoon instruction. No matter how vigorous the nervous system may be, the brain ought not to be taxed while in the process of growth, while digestion is going on. Under unfavorable external conditions abnormal fatigue will prevail throughout the whole afternoon, and in the course of time the brain will become unfit for mental work. Afternoon instruction is therefore absolutely unhygienic. Teachers are unanimous in pronouncing afternoon mental work a failure in the majority of cases. One long morning session, with a number of recesses, ought to suffice. Let that instruction be rational, let it be largely object teaching; do not overfeed children with grammar, which presupposes mastery of speech and the power of developed thought. The brain-cells required for such work are as yet imperfectly developed, and the natural result is a mechanical memorizing resembling the training of a parrot. How different the effect of the introduction of the various mechanical arts. They appeal at once to the various organs of sense, and through them to the intellect. Large playgrounds should be provided in every city, and every schoolhouse should be joined by a large roof-covered area, equipped with the most important apparatus necessary for physical training for both summer and winter. The authorities ought to see to it as much as possible that each child is given the opportunity to develop its individual inclinations, for in the nature of the case, and necessarily so, class-room work has a leveling tendency; its laws are those of a liberal, sometimes even of an illiberal, despotism; a child becomes one of a dozen and nothing more, and the only virtue practiced is that of obedience, which it should have acquired at home. On the play-ground the development of individuality ought to be given a chance, but always under the control of the wiser parent or teacher.

---

### THE USE OF MIDWIFERY FORCEPS.

J. M. Munro Kerr, M.B., C.M., F.F.P.S., Glasgow (*Lancet*, September 24th, 1898), enters a protest against the present day teaching of applying the blades of the obstetric forceps with exclusive reference to the transverse diameter of the maternal pelvis:

"The author takes exception to the general teaching of the present day in this country that, so long as the blades of the forceps are applied transversely as regards the maternal pelvis, it does not matter, or, at least, it need not be considered how they are disposed as regards the head of the child. This is a departure (a Continental importation) from the older teaching of obstetricians in this country, who from Smellie's time until quite recently laid great stress upon the importance of applying the blades, both of the straight and double-curved forceps, with a due consideration not only of the pelvis, but also of the diameters of the fetal head. The present day teaching is without doubt largely due to the writings of Dr. Barnes, who, in his 'Lectures on Obstetric Operations,' maintains that while with the straight forceps 'the head determines the manner of applying it,' continues, 'but with long forceps it is the pelvis that rules the application. The position of the head may be practically disregarded. We, of course, are only concerned here with the double-curved forceps.

"Writing on the application of the forceps, Dr. Playfair and Dr. Galabin, in their respective text-books on midwifery, express themselves as follows: (I quote from them because their textbooks are the two most popular in the various medical schools of this country.) Dr. Playfair writes: 'It is admitted that in the high-forceps operation the blades must be introduced in the transverse diameter of the pelvis without relation to the position of the head.'

"Dr. Galabin writes: 'Accordingly, it is now the usual plan, even if straight forceps are used, not to pay much regard to the position of the head, but apply the blades, one at each side of the pelvis. In the case of the long curved forceps, it is generally taught that the blades should be applied at the sides of the pelvis without regard to the position of the head. The position of the head should, however, be exactly determined in the first instance, not so much that any great difference in the position of the blades should be aimed at in consequence, but rather that,' etc.

"It may be taken, then, from these quotations, that the general teaching and practice in this country in applying forceps is to consider only the pelvis of the mother, and to apply the blades always in the transverse diameter. Now, I consider such teaching unwise and incorrect. It is unwise, because it tends to encourage carelessness from its lack of exactness. In applying the blades of the forceps both the child's head and the pelvis of the mother should be considered; and when an operator commences to exert traction, not only should he know how the blades are lying with regard to the maternal pelvis, but also how they are lying with regard to the head of the child. It is also incorrect.

Dr. Playfair says, speaking in vindication of the teaching of applying the blades of the forceps always in the transverse diameter of the maternal pelvis, that he agrees with Dr. Barnes, who points out that "do what we will, and attempt as we may to pass the blades in relation to the child's head, they find their way to the sides of the pelvis.'

"But such a statement as the above—and please observe it is a very generally expressed one—is, if one only thinks of it for a moment, tantamount to saying that the forceps is an instrument over which we have not complete control; that one operator will grasp the head as well as another; that all the operator has got to do is to introduce one blade to one side and the other to the other side of the pelvis, when the blades pass beyond his control and adjust themselves to the fetal head in the best direction possible. But further proof of the incorrectness of the statement and general teaching will be found clinically. It will, I think, be admitted by all who have had much experience in obstetric practice that a head which was causing very great difficulty in extraction may sometimes be easily delivered by altering the grasp of the forceps. Now that is not because with the previous grasp the operator was exerting traction in a wrong direction, for before readjusting the instrument every operator would make attempts at traction in every possible direction. Neither is it because, on the first occasion, the blades of the forceps were not applied in the transverse diameter of the pelvis. The reason is simply that certain grasps of the head are better than others. In each case there is one grasp better and safer for mother and child than all others. The better grasp, however, is not likely to be secured by applying the blades of the forceps to the sides of the pelvis, and trusting to the chance of their adapting themselves to the most favorable diameter of the head. Much more likely is it to be obtained by applying the blades in a certain diameter of the child's head which, after due consideration of the case, the operator believes to be the best.

"Now a word or two with regard to the application of the forceps at the outlet, at the brim, and in flat pelves.

"1. At the outlet—Here, the long axis of the head having rotated into the conjugate diameter of the pelvis, the blades can as easily be applied with reference to the diameters of the fetal head as with reference to the diameters of the maternal pelvis. When straight forceps were in use for this operation they were always applied with reference to the child's head, and Dr. Barnes still recommends that method of applying them, as may be seen from the quotation I have already given.

"2. At the brim—Here, in spite of all one's efforts, it is nearly always impossible to grasp the fetal head bi-parietally. The grasp must be more or less oblique in most cases. What I maintain, however, is that by considering the fetal head and applying the blades deliberately to it as nearly as possible in the bi-parietal diameter a less oblique grasp is obtained, one which will less often slip and one which will require less frequently to be altered than if the blades as recommended are simply introduced into the transverse diameter of the pelvis, traction exerted, and the grasp of the head never considered.

"3. In flat pelves—In such cases it is of the very highest importance that the fetal head be deliberately grasped. In flat pelves, when the long axis of the fetal head lies very nearly in the transverse diameter of the pelvis, the application of the blades of the forceps in the transverse diameter of the pelvis without reference to the head results in one of the three following grasps: (*a*) the head is grasped directly in the antero-posterior diameter—a very rare occurrence; (*b*) it is grasped very nearly but not quite in the antero-posterior diameter, also a rare occurrence; in such cases there is often the mark of the tip of one blade just above the eye; and (*c*) the head is grasped quite obliquely, one blade is to the side of the occiput and the other blade to the side of the forehead. This is by far the most usual grasp obtained.

"What I maintain here is that if one applies the blades of the forceps as is now generally taught, one cannot tell which of the above three grasps of the head one will get; it is entirely a matter of chance as far as the operator is concerned. Surely there is something wrong in a teaching into which the element of chance enters, especially when it is quite unnecessary that it should do so. To avoid it the operator has simply first to satisfy himself as to whether in a particular case it is better to grasp the head antero-posteriorly or obliquely, and then deliberately to apply the blades to the child's head in the one or the other direction. That deliberate grasping of the child's head was the general teaching of obstetricians up till thirty years ago, only they, of course, approved only of the 'oblique grasp,' as they considered the antero-posterior one very dangerous to the child. Into the question of which of the two 'grasps' is the better I will not enter at present. I will only say that in certain cases the one, and in certain cases the other, appears to me the safer for child and mother, and that the indications for the one or the other will be found in the variety of flat pelvis one is dealing with.

"In conclusion, I would only add that, although the teaching I have been here condemning is very general, I know it is not universal. Dr. Milne Murray, in his most able paper at the annual meeting of the British Medical Association, the other day, referred to the matter and advocated a deliberate grasp of the head in flat pelvis, and other teachers in Edinburgh and elsewhere, do the same."

---

### AMBULANCE CLASSES—A BEAUTIFUL FAD.

With the coming of the summer the voice of the lecturing physician on "First Aid to the Injured" was silent in the land; and evidently with his devotees it is out of hearing, out of mind, otherwise some of his precepts would have been put into practice when occasion required, as it has frequently during the past few months. Perhaps, however, we are censuring without just cause, so we pause to ask a question: "What has been taught in these 'Ambulance Classes?'" Has, for instance, the rescuing and the resuscitating of the drowning been made the subject of lecture and study? If so, passing strange, is it not, that any should perish, judging by the enormous attendance accredited to these classes. In fact, half the population seems to have been seized with a longing for a wider sphere of usefulness. Pity 'tis, that the fancy has taken such a morbid direction. The public press has kindly chronicled long lists of those who have "passed the necessary examinations." Here may we venture another question: "What are the graduates qualified to do?" Are their qualifications simply to stick a pin in a bandage, without causing the oftentimes ungrateful subject of their ministrations to "see stars?" One would be inclined to pity the long-suffering physicians who have so courteously given their time to a cause so apparently useless. Are these classes, that have been the fad of the past two winters, needed? They have been attended alike by all grades of society, from the ladies of Toronto, the Y. M. C. A. men, etc., down to the housemaids. Perhaps the wisest thing the physician can do is to bow the misguided ladies back into the social circles, which they so gracefully adorn, or pray for a new fad to become epidemic.

As for these Guild classes, what is the use of physicians bothering themselves to talk to housemaids? Little can be accomplished except a cheapening of the dignity of the medical men themselves. As for the young men mentioned, possibly better results might be obtained, as few "boys grown tall" trouble themselves to listen to lectures or learn anything unless they intend to make some practical use of the knowledge attained. However, we think these classes are not needed, and too often the truthfulness of the good old maxim has been proven—"Little knowledge is a dangerous thing."

We do not wish to censure sweepingly, as there are several classes in our community to whom lectures upon "First Aid to the Injured" should be given; for instance, railway men, steamboat employees, and all such as are exposed by reason of their occupation to unusual danger.

Nevertheless, we are firmly of the opinion that, thoughtlessly, perhaps, a number of our physicians are lowering the standard of the medical man by freely imparting to the laity too much medical knowledge. If this fad continues it may soon come into vogue to consult one's patients as to what they would prefer as an antidote to poison, or perhaps, in a case requiring surgical treatment, a serious argument may occur between physician and patient, the latter feeling his superior knowledge, owing to his having been a "grad" of the ambulance class of the winter of '89.

As physicians, let us remember where we put ourselves we must stay. Already the wise worldlings engaged in other professions are laughing at our prodigal liberality; so let us, ere it is too late, keep the knowledge that it has taken years of study to acquire, imparting it cheerfully only to those who intend to espouse our noble calling as a lifework. We have already cast our bread to feed the stranger, for have we not given, with scarce a protest, the name "Doctor," to be framed, chromo-like, to enhance the barndoors of quackdom?—*Canadian Journal of Medicine and Surgery.*

---

### PHYSICIAN'S ACCOUNTS AND THE LAW.

A recent issue of the *Philadelphia Polyclinic* contains an article by Dr. W. G. Porter and an editorial by Dr. S. Solis-Cohen on

"Does a Physician's Book-Account Constitute a Legal Claim," which is most timely, important and surely worthy the notice, if not the deepest concern of the general profession. The case in question was briefly, a physician, living in same house with patient, presented a bill upon the death of patient against the estate. The lawyer representing the estate said that the physician must prove his bill to the satisfaction of the auditing judge. It was asserted that many of these visits were social. The physician had kept an ordinary visiting list and ledger.

"The judge refused to allow the attending physician to go on the stand and swear as to the truthfulness of his account, but ruled that the bill must be proved by the evidence of a third party, or the production on his part of what he called a book of original entry—that is, a book in which each visit is entered with the amount charged therefor, so much for a day visit, so much for a night visit, so much for a vaccination, so much for a confinement, etc. As under this ruling neither the visiting list nor ledger are considered books of original entry."

Again:

"Within a few days the same judge, in the same court, if he is correctly reported, rejected the claim of another physician for professional services to a decedent, from May 2, 1888, to November, 1893—596 visits, at $1.50 each, $894. 'All of this claim, prior in date to May 11, 1893, was barred by the statute. How many visits were paid between May 11, 1893, and November 27, 1893, was not shown. The physician's books were produced, but they were not books of original entry, in any technical sense, and were clearly inadmissible.' And thus, after five years and a half of faithful service, the physician received his reward--'nothing'—and this is law, if it is not equity."

These two extracts need no comment to show how few physician's accounts could be collected if this ruling of the courts will stand. Physicians are proverbially poor bookkeepers and collectors, but if this is the condition imposed by the law it behooves all to "turn over a new leaf."

In the same journal according to Mr. A. F. Curtis, of the Philadelphia bar:

"In order to make an entry of charge admissible as evidence it must be properly dated, be made against a definite person, show a definite service to that person, or to one for whom he is legally responsible, and must have stated terms of money a definite charge for the service. Moreover, the entry must have been made not later than the day after the service was rendered."

To live up to these strictures will impose more work or more expense on an overburdened profession, yet in these days when people delight in and purposely boast of "beating" the doctor, a word of warning is necessary that we may protect ourselves in substantiating honest claims and due recompense for labor and services often too freely and cheerfully rendered.

---

### THE URINE AS A DIAGNOSTIC FACTOR.

Dr. Kernode (*Tri-State Med. Jour.*, p. 369, August, 1898) concludes an article with the above title, with the following succinct rules, first formulated by Dr. Formad and verified by many investigators:

1. Sediment in the urine has no significance unless deposited within twenty-four hours.

2. Albumen in the urine does not indicate kidney-disease unless accompanied by tube-casts. The most fatal form of Bright's disease—contracted kidney—has little or no albumen.

3. Every white crystal in urine, regardless of shape, is a phosphate, except the oxalate-of-lime crystal, which has its own peculiar form; urine alkaline.

4. Every yellow crystal is uric acid if the urine is acid, or a urate if the urine is alkaline.

5. Mucous casts, pus, and epithelium signify disease of the bladder (cystitis) or other parts of the urinary tract, as determined by variety of epithelium.

6. The urine from females can often be differentiated from the urine of males by finding in it the tesselated epithelium of the vagina.

7. Hyaline casts (narrow), blood, and epithelial casts signify acute catarrhal nephritis. There is much albumen in this condition.

8. Broad hyaline casts and epithelial dark-green granules and oil-casts signify chronic catarrhal nephritis. At first, much albumen; later, less.

9. Hyaline and pale granular casts, and little or no albumen, signify interstititial nephritis.

10. Broad casts are worse than narrow casts, for the former signify a chronic disease.

11. The urine should be fresh for microscopical examination, as the micrococci will change hyaline casts into granular casts, or devour them entirely in a short time.

12. Uric acid may, in Trommer's test for sugar, form a peroxide of copper, this often misleading the examiner into the belief that he has discovered sugar. Thus when urine shows only sugar, other methods of examination must be used—preferably the lead test.

13. The microscope gives us better ideas of the exact condition of affairs in examination of urine, than the various chemical tests.

R.

---

### UTERINE COUGH.

Schaeffer (*Centralbl. für Gynäkologie*, 1893, No. 31) concludes as follows:

"1. In those predisposed, such as neuropaths and sufferers from genital diseases (especially during menstruation and pregnancy), cough may at times be induced by isolated contact with the *fornix vaginæ*.

"2. In those so predisposed, pathological processes which involve the broad ligaments, and especially Douglas' pouch, may cause reflex cough, just as they cause reflex acne and hyperemesis. In the latter conditions abnormal fermentation and auto-inoculation are probably also present.

"3. In those predisposed to cough as above stated, irritation of the lower third of vagina and vulva can occasion only local reflexes—nothing remote, such as a cough, can occur.

"4. Uterine cough is produced by irritation, on one side, of the utero-vaginal fibers of the hypogastric plexus which supply the fornix vaginæ and collum uteri, and, on the other side, the spermatic plexus, hemorrhoidal nerves and ganglia imbedded in the broad ligament which supply the fundus uteri and ovaries.

"5. Irritation which affect the nervous pudendus are at first localized in their reflex effects.

"6. Reflex phenomena may be (*a*) essential physiological reflexes in remote motor and vaso-motor territories, which, through the neuropathic basis are easily set in motion; (*b*) radiation in the case of neuropaths, where resistance is weakened; (*c*) irregular radiation in high degrees of neuropathy.

"7. Cases of tuberculous habitus, or predominance of stomach symptoms, play a separate part in the genesis of nervous cough.

"8. Local treatment, especially by pessaries, acts promptly when pathological conditions are complicated, as in the case of retroflexion and prolapse, etc."

---

### PSORIASIS.

J. V. Shoemaker (*Pa. Med. Jour.*, August, 1898) observes that psoriasis of the scalp does not cause baldness. The causes of this disease are from within, and can generally be traced to some morbid condition of the blood or disturbance of the nervous system. The author has found that the rheumatic or gouty diathesis frequently underlies an attack. Local measures are of secondary importance, and internal management depends upon the origin in each case. In all cases the patient must be placed under the most favorable hygienic conditions. If the cause lie in disturbance or lesion of the nervous system remedies exercising a beneficial and nutritive influence upon nerve-tissue are indicated. Electricity, static or galvanic, will be found valuable. Massage, moderate exercise, baths, diaphoretics, and the salicylates, iodides and sulphur in cases depending upon rheumatic diathesis are appropriate agents. The bowels and kidneys must act normally. Drugs which disarrange digestion will do more harm than good. Locally, loose the scales by the use of water, oil, a poultice or a general bath. Ointments of tar, carbolic acid, creosote, turpentine, sulphur or salicylic acid may be used. Chrysobarin and pyrogallol are powerful agents, but have conspicuous disadvantages. G.

### MATERIA MEDICA AND THERAPEUTICS.

### FASTING IN THE TREATMENT OF INFECTIOUS DISEASES.

De Domenicis (*Wiener Med. Presse*, May 1st, 1898) is a strong believer in the old doctrine of fasting in fevers, and his zeal has earned for him the name of the "hungry doctor" among the laity of Naples. Taking pneumonia as an example, he has made a study of 140 cases from this point of view. A specific virus and exposure to cold are not enough in themselves to cause pneumonia. The third factor is a bad state of the gastro-intestinal canal, and a resulting condition of auto-intoxication which lowers the vitality of the patient, and increases the virulence of the pneumococcus. The author, therefore, washes out the stomach in these cases, and then administers intestinal disinfectants, and this procedure is followed by a fast of several days, or even of a week, in certain cases. In other words, the author believes that the deadly agency in pneumonia comes from the specific toxins as much as from products in the intestinal canal. It would appear that the author does not use his fasting therapeutics unless there is some special evidence of gastro-intestinal auto-intoxication. He mentions arthritis as another form of disease eminently suitable for fasting.

---

### EXTRACT OF SUPRA-RENAL CAPSULE.

Corona (*La Riforma Medica*, May 18th, 1898) gives the results of his investigations:

"I. Supra-renal extract is toxic, and the constituents are of a nature opposed to one another.

"II. It is lethal in small, repeated doses.

"III. It is cumulative in action.

"IV. It acts upon the heart, first exciting, and then paralyzing it.

"V. It has no notable action upon the formed elements of the blood.

"VI. After time its action modifies the pigment of the skin (in frogs, etc.), causing it to clear up, probably through its operation upon the chromatophores which contract strongly.

"VII. Extirpation of both capsules is fatal."

---

### RELATIONSHIP BETWEEN THE MEDICINAL DOSE AND THE PECULIARITIES OF THE GROWING ORGANISM.

Troitsky (*Jahrbuch fur Kinderheilhunde,* 1898, April 15th), after a very elaborate study of this subject from every point of view, gives his conclusions as follows:

1. Our increasing knowledge of the anatomy, physiology and chemistry of the growing organism gives the practitioner the right to demand similar progress in posology.

2. New data are recognized in the etiology, course, diagnosis and prognosis of disease, and cannot be ignored in the treatment.

3. The peculiarities of the circulatory organs, blood, digestive organs, nervous system, skin, body weights, should furnish data for proper dosage.

4. The following substances have not been particularly studied with respect to the growing organism: Preparations of iron and lime, alkalies and acids, salts of alkalies, sodium benzoate and salicylate, the expectorants, quinia, potassium iodide and phosphorus.

5. The number of years must be revised as a guide to dosage, because metabolism, resorption and excretion, rapidity of circulation, specific gravity, blood pressure, etc., do not always bear a constant relationship to age.

6. The increments of growth influence the increments of dosage in such way that the latter must be diminished at various periods.

7. Age, body weights and rate of growth are the three factors from which an arithmetical mean should be determined in selecting a dose.

---

THE oil of sassafras will destroy all varieties of pediculi and their ova with a single application. Care must be taken to prevent its coming in contact with mucous membranes. Any burning from this cause can be allayed in a few minutes by pouring on olive oil.—*Medical Brief.*

---

### INTERNAL TREATMENT OF URETHRITIS.

By Frank Lydston, M.D., *American Text-Book of Genito-Urinary Diseases:*

| | | | |
|---|---|---|---|
| ℞ | Liq. potassii | 40 | (ʒi.) |
| | Bals. copaibæ | 30 | (℥i.) |
| | Ol. gaultheriæ | 65 | (m x.) |
| | Ext. glycyrrhizæ, fl | 15 | (℥ss.) |
| | Saccharin | 95 | (q. s.) |
| | Muc. acaciæ, q. s. ad | 120 | (℥iv.) |

Sig.: A teaspoonful every two or three hours.

---

### FOR PRURITUS.

| | | | |
|---|---|---|---|
| ℞ | Menthol | 4 | (ʒi.) |
| | Cerat. simp | 60 | (℥ii.) |
| | Ol. amygdalæ dulc | 30 | (℥i.) |
| | Ac. carbolic | 4 | (ʒi.) |
| | Pulv. zinc. oxid | 60 | (℥ii.) |

After cleansing the parts apply night and morning.—*C. B. Kelsey* (*Philadelphia Medical Journal*).

---

FOR hoarseness in singers and speakers Botey commends the following:

| | | | |
|---|---|---|---|
| ℞ | Cocain. hydrochlor | 1 | (gr. xv.) |
| | Strich. sulph | 04 | (gr. ¾.) |
| | Aq. destillat | 90 | (℥iii.) |

Spray throat.

And:

| | | | |
|---|---|---|---|
| ℞ | Cocain. hydrochlor | 04 | (gr. ¾.) |
| | Tr. aconiti | 65 | (m. x.) |
| | White sugar and marshmallow | | (q. s.) |

Ft. pastillas No. lxxx. S. To dissolve in the mouth.—*Gaillard's Medical Monthly.*

### DYSPEPSIA CURE RECOMMENDED BY COE.

| | | | |
|---|---|---|---|
| ℞ | Pulv. rhei | 8 | (ʒii ) |
| | Ext. gentian, fl | 12 | (ʒiii.) |
| | Aq. menth. pip | 225 | (℥viss ) |
| | Sod. bicarb | 24 | (ʒvi) |

M. Sig.: A teaspoonful half an hour before meals.—*Louisville Medical Journal.*

---

### TREATMENT OF TAPEWORM.

The following is credited to E. Chamberlin:

| | | |
|---|---|---|
| ℞ | Alcohol containing 10 per cent. of chloroform | 8 parts. |
| | Rectified oil of turpentine | each 4 parts. |
| | Ethereal extract of male fern | |
| | Glycerin | 15 parts. |

M. Half a teaspoonful to be taken every hour. Before beginning the use of this mixture the patient should take castor oil or magnesium sulphate, and as soon as a purgative effect is produced, the mixture may be taken. For very young subjects, for example, children two years old, the formula may be modified as follows:

| | | |
|---|---|---|
| ℞ | Alcohol containing 10 per cent of chloroform | |
| | Rectified oil of turpentine | each 2 parts. |
| | Extract of male fern | |
| | Glycerin | 15 parts. |

M. Sig.: A teaspoonful every hour.

---

### SOLOMON'S MIXTURE FOR INFANTILE CONVULSIONS.

| | | | |
|---|---|---|---|
| ℞ | Moschi | | 5 (gr. vii.) |
| | Gum acacia | 2 | (ʒss.) |
| | Aq. fœniculi | | |
| | Syr. aurant. cort | āā 30 | (℥i.) |

M. Sig.: A teaspoonful every hour or two.—*New York Medical Journal.*

---

### INJECTIONS DURING THE ACUTE STAGES OF URETHRITIS FOR A SEDATIVE ASTRINGENT.

| | | | |
|---|---|---|---|
| ℞ | Plumbi acetatis | | 26 (gr. iv.) |
| | Vini opii | 8 | (ʒii) |
| | Aquæ rosæ, q. s. ad | 15 | (℥ss.) |

Sig.: Inject three times daily.

This is to be followed by a bichloride of mercury injection (solution 1 to 15,000), in combination with a small amount of glycerin.

### IN THE LATER STAGES OF GONORRHEA.

| | | | |
|---|---|---|---|
| ℞ | Terebinth. alb | 4 | (ʒi.) |
| | Res. podoph | | 03 (gr. ss.) |
| | Camphor-monobrom | 4 | (ʒi.) |

M., et ft. pill No. 30.

Sig.: One pill four times a day.

---

### TO ALLAY CHORDEE AND SEXUAL EXCITABILITY.

| | | | |
|---|---|---|---|
| ℞ | Ext. ergotæ, fl. | 1 | (m xv) |
| | Tr. gelsemii | | 32 (m v.) |
| | Potassii bromidi | 1 | 32 (gr. xx.) |
| | Tr. hyoscyami | 2 | (ʒss.) |
| | Syr. aurantii, q. s. ad | 15 | (℥ss.) |

Sig.: To be taken at bedtime.

---

### DURING THE STATIONARY STAGE OF URETHRITIS THE FOLLOWING IS MORE SERVICEABLE.

| | | | |
|---|---|---|---|
| ℞ | Zinci sulph. (acetat.) | | (gr. xii.) |
| | Morph. sulph | | 65 (gr. x.) |
| | Glycerin | 30 | (℥i.) |
| | Aquæ rosæ | 90 | (℥iii.) |

Sig.: Injection.

---

FOR boils L. Duncan Bulkley commends the following for local application:

| | | | |
|---|---|---|---|
| ℞ | Acid. carbol | | 3-.65(gr. v.-x.) |
| | Ext. ergot | 4–8 | (ʒ i–ij.) |
| | Pulv. amyli | 4 | (ʒ ij.) |
| | Zinci. oxidi | 4 | (ʒ ij.) |
| | Ung. aquæ rosæ | 30 | (ʒ viij.) |

---

### OINTMENT FOR VARICOSE ULCER.

| | | | |
|---|---|---|---|
| ℞ | Ac. carbolici | 2 | (ʒ ss.) |
| | Ac. borici | 10 | (ʒ iiss.) |
| | Camphoræ | 8 | (ʒ ii.) |
| | Ichthyol | 20 | (ʒ v.) |
| | Ol. amygdalæ dulc | 10 | (ʒ iiss.) |
| | Ung. zinci oxidi | 105 | (℥ iiiss.) |

M. Sig.: For external use.—*Gaillard's Medical Journal.*
